# Resting state in Alzheimer's disease: a concurrent analysis of Flash-Visual Evoked Potentials and quantitative EEG

**DOI:** 10.1186/1471-2377-12-145

**Published:** 2012-11-28

**Authors:** Antonio Tartaglione, Luciano Spadavecchia, Marco Maculotti, Fabio Bandini

**Affiliations:** 1 Dept of Neurology, Ospedale S. Andrea, La Spezia, Italy; 2 Dept of Neurology, Ospedale S. Paolo, Savona, Italy; 3 Dept of Neurology, DINOG, University of Genova, Genova, Italy; 4 Istituto di Biofisica, CNR Genova, Genova, Italy; 5 Dept. Scienze Neuroriabilitative - Casa di Cura Privata del Policlinico s.p.a., Milano, Italy; 6 Centro “La Missione” – Sarzana, La Spezia, Italy

**Keywords:** Alzheimer's disease, F-VEP, q-EEG, Resting state, Eye-closed, Eye-open, Dementia

## Abstract

**Background:**

To investigate to what extent Alzheimer's Disease (AD) affects Resting State activity, the possible impairment of independent electrophysiological parameters was determined in Eye-open and Eye-closed Conditions. Specifically, Flash-Visual Evoked Potential (F-VEP) and quantitative EEG (q-EEG) were examined to establish whether abnormalities of the former were systematically associated with changes of the latter.

**Methods:**

Concurrently recorded F-VEP and q-EEG were comparatively analysed under Eye-open and Eye-closed Conditions in 11 Controls and 19 AD patients presenting a normal Pattern-Visual Evoked Potential (P-VEP). Between Condition differences in latencies of P2 component were matched to variations in spectral components of q-EEG.

**Results:**

P2 latency increased in 10 AD patients with Abnormal Latency (AD-AL) under Eye-closed Condition. In these patients reduction of alpha activity joined an increased delta power so that their spectral profile equated that recorded under Eye-open Condition. On the opposite, in Controls as well as in AD patients with Normal P2 Latency (AD-NL) spectral profiles recorded under Eye-open and Eye-closed Conditions significantly differed from each other. At the baseline, under Eye-open Condition, the spectra overlapped each other in the three Groups.

**Conclusion:**

Under Eye-closed Condition AD patients may present a significant change in both F-VEP latency and EEG rhythm modulation. The presence of concurrent changes of independent parameters suggests that the neurodegenerative process can impair a control system active in Eye-closed Condition which the electrophysiological parameters depend upon. F-VEP can be viewed as a reliable marker of such impairment.

## Background

It has been reported that in Resting State [1], Eye-open and Eye-closed Conditions correspond to distinct patterns of activation [2-4], outlining different cortico-subcortical network systems [5]. In the light of recently postulated network degeneration hypothesis [6,7] it is reasonable to consider the possibility that Alzheimer's Disease (AD) affects separately either system.

Some data derived from analysis of Flash-Visual Evoked Potential (F-VEP) and quantitative-EEG (q-EEG) frequency spectra seem to support this assumption.

In normal Conditions the two modalities are independent from each other and present a different behaviour in Resting State. F-VEP latency, measured at its P2 component peak, does not differ when moving from Eye-open to Eye-closed Condition [8] whereas EEG activity does, as established since Berger's first observation (1929).

AD may interfere with both modalities in Eye-closed Condition. As a matter of fact the increase in F-VEP latency, often reported [9-12] in AD, has been mainly referred to this Condition [8]. By the same token, AD is characterized by a reduction of normal dominant posterior EEG rhythm (for reviews see [13-15]) with a significant decrease of alpha power [16,17] in Eye-closed Rest State.

Since changes in both modalities occur under the same Condition, it is possible that they reflect the involvement of a neural system active in Eye-closed Rest State which F-VEP and q-EEG modulations separately depend upon. If the two modalities depend on a network active in Eye-closed Condition, both should be affected by lesion of this system. Therefore, AD patients ought to present concurrent changes of F-VEP and q-EEG under Eye-closed but not under Eye-open Condition.

The data so far available are not suited to confirm such a hypothesis since F-VEP changes and EEG defects have been separately investigated in different AD studies. All underlined the individual variability of each parameter [8,18]. No one investigated their parallel variations in specific Conditions and in single patients. Hence it is not clear whether variations in F-VEP are associated to changes in q-EEG.

The present study was carried out to determine what is the likelihood for F-VEP changes to be associated with modifications of quantitative EEG (q-EEG) in AD patients and to ask whether the pattern of change is the same in the whole group. The result would also provide an useful criterion to identify a group of patients sharing the same functional damage and, possibly, the same distribution of damage [19].

## Methods

### Subjects

The study was carried out on 39 subjects, 17 Controls (10 men and 7 women) and 22 AD patients (14 men and 8 women) consecutively admitted to the wards of the Department of Neurology of the University of Genoa. All patients agreed to participate in the study with full knowledge of the nature of the research. After a complete explanation of the study, written informed consent was obtained from each subject. The Medical Review Ethics Committee of the Department of Neuroscience, Ophthalmology and Genetics - University of Genova approved the study.

Patients affected by dementia, as defined by DSM-IV, had to meet the following criteria: a) diagnosis of Probable AD according to the definition of NINCDS-ADRDA [20]; b) degree of cooperation sufficient to carry out a Pattern-Visual Evoked Potential (P-VEP) sequence.

With a score of less than 7 at the Hachinski Ischemia Scale [21], AD patients had no evidence of focal abnormalities at CT or MR scans. Other causes - infectious, toxic, metabolic - of dementia were excluded. Controls who reported no past history of neurological or psychiatric disease were patients in the Department of Internal Medicine of the University of Genova. Information about patient's history, review of medical chart and clinical interview allowed to exclude the presence of psychiatric disturbances in Controls who underwent neurological and ophthalmological investigations to exclude the presence of specific signs or symptoms. Pupil reactivity and intraocular pressure were normal in all the patients. Visual acuity had to be no less than 15/20*.* All the patients had normal P-VEP parameters. Cognitive changes were assessed by Mini-Mental State Examination (MMSE) [22,23] and Dementia Rating Scale (DRS) [24,25]. Patients did not differ from Controls with respect to age (t: 1.01; df: 37; ns) or educational level (t: 0,17; df: 37; ns), while they did with respect to MMSE (t = 11,7; df: 37; P < 0.001) and to DRS (t = 15,48; df: 37; P < 0.001) scores.

### Procedure

q-EEG and Visual Evoked Potentials (VEPs) were recorded in a quiet room, with the subject awake, seated on a comfortable chair under continuous control. In order to avoid interference between photic stimulation and Resting State activity, F-VEP was recorded separately from EEG, in the same session.

#### Visual evoked potentials

VEP was recorded in 78 eyes, 34 in the Control Group and 44 in the AD one. P-VEP and F-VEP were elicited by monocular stimulation. The EEG signals, picked up with 3 silver-silver chloride electrodes placed at Oz, O1 and O2 and referred to a common electrode (Cz), were amplified at bandpass 0.53-100 Hz and averaged. VEPs evoked during large EEG oscillations due to head and/or ocular movements were discarded.

P-VEPs were generated by pattern reversal at 1 Hz frequency, using a checkerboard subtending 10° with a square profile elements of 55' side and a contrast of 96%. No less than 256 stimuli were delivered to each eye and P100 latency was measured as the peak of the PVEP major positive component. P-VEPs were considered abnormal if their P100 latency was greater than 118 msec. and their interocular difference more than 8 msec.

F-VEP was generated at 1 Hz frequency by a white flash whose energy was 0.5 joules, corresponding to 10 lux of illuminance at 30 cm distance. The analysed epoch, i.e. 300 msec, allowed to measure the latency of P2 and N3 components of FVEP, but the study was restrained to the first component due to uncertainties in identifying the N3 peak. Peaks latencies, evaluated by two independent observers, were accepted if measures differed by less that 5 msec, otherwise components were considered “missing” and discarded. Analysis of F-VEP was done on three channels, Oz, O1, O2. Since data from O1 and O2 did not differ from those of Oz our report will be restricted to the latter.

Flash was presented under Eye-open and Eye-closed Conditions. The unstimulated eye was covered with an opaque patch and the patient was asked to hold his/her hand over the patched eye.

**Figure 1:**
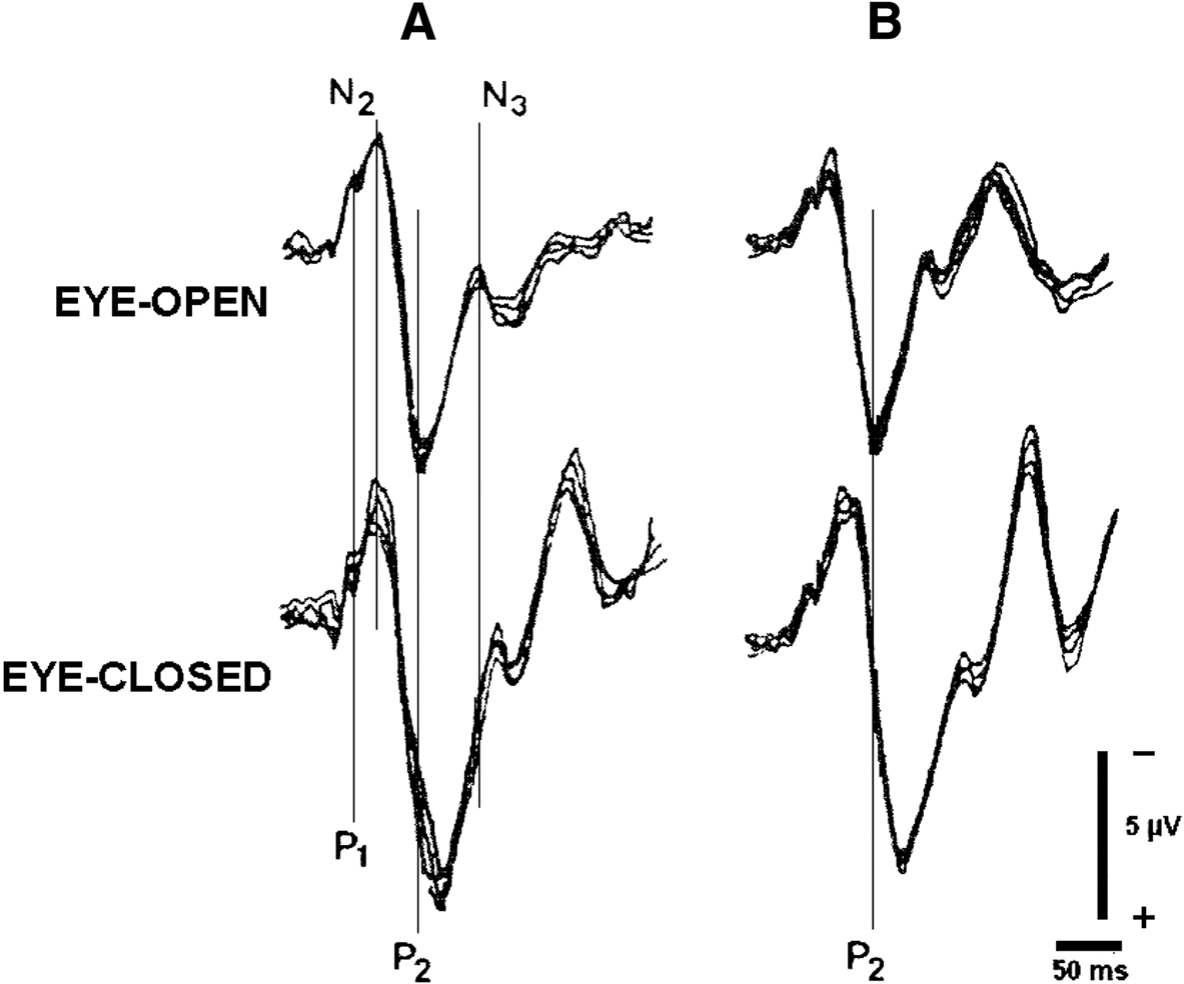
**F-VEPs recorded in an AD-AL patient from left (A) and right (B) eye were averaged from 4 separate blocks of 64 trials.** In **A** the distribution of components within waveform are presented. Latency of P2, as shown in **B**, is the parameter taken into account in the study.

As shown in Figure 1, no less than 4 blocks of 64 trials each were collected for Eye-open and Eye-closed Conditions, in random sequence. The blocks were averaged and latencies were measured on the final result. Each session lasted no longer than 30 min.

9 patients presented a "missing" component on both eyes in one Condition, mainly the Eye-open one (see Results). Among the remaining 30 patients P2 latency could be identified in both eyes of all the Controls under Eye open Condition and in 9 of them under Eye-closed one (Additional file 1: Table S1). P2 latency could be identified in both eyes in 17 out of 19 AD patients under either Rest Condition. Missing values came from different patients (Additional file 1: Table S1).

Whenever present, individual P2 latencies of OD and OS significantly correlated with each other both in Eye-closed Condition (coefficients of correlation: r = 0,85 in Controls and r = 0,93 in AD patients) and in Eye-open one (coefficients of correlation: r = 0,90 in Controls and r = 0,91 in AD patients). Mean latencies did not differ in two eyes within group and conditions (Additional file 1: Table S1) confirming the result of correlation. This enabled us to average OD and OS latencies in order to yield a single value for each patient and condition.

#### q-EEG

A standard 16-lead EEG was acquired as a continuous signal for no less than 30 minutes, visually inspected for current interpretation and detection of artifacts, and stored for off-line analysis. All scalp electrodes were referenced to linked ears, and a site between Fpz and Fz was used as ground. Since the study was meant to examine mainly variations in posterior rhythms, the analysis was limited to 6 channels i.e. O1, O2, C3, C4, T3, T4. Had we extended the analysis to the whole set of channels (16) we would have found a mass of data difficult to interpret. In particular it was felt that such an extension might mask slight but significant results, due to topographical changes in different regions and in opposite directions. Electro-oculogram was also recorded from tin cup electrodes placed above and below the left eye and on the outer canthus of each eye. Impedance was kept below 5 kΏ for all the electrodes. 10 minutes signals from the computer stored EEG were collected for each Condition, amplified, sampled by a 16 bit A/D converter with a sampling rate of 1024 Hz. and digitally filtered in the frequency range 1-100 Hz. No less than 27 blocks of artifact-free 2,5 s-long epochs were selected off-line by visual inspection for Eye-open and Eye-closed Conditions.

### Data analysis

Individual P2 latency was defined as the mean of the values recorded from the two eyes in Eye-open and in Eye-closed Conditions. In case of "missing" P2, the value evoked from the fellow eye was taken into account.

In order to avoid that the overlap of highly variable measures might mask possible variations among groups or between Conditions, the difference between P2 latencies recorded under Eye-open and Eye-closed Conditions was computed. The difference was considered abnormal if it outweighed the normal cut-off of 14,8 msec, i.e. the limit of one-tailed confidence interval in mean difference of Controls estimated with probability p<0,001 (t_α0,001_, df 11 = 4,1).

Based on Rodriguez et al.'s [26] observation, q-EEG recordings obtained under either Condition were separately analysed by performing a Fast Fourier Transform (FFT) with a resolution of 0.39 Hz in the frequency domain between 0.39 Hz and 32 Hz. The absolute values of all epoch spectrum frequencies were averaged to compute the Mean Power Spectrum (MPS) of each channel for Eye-open and Eye-closed Conditions. MPS was partitioned into 4 component bands, i.e. delta: 0.39 – 3.90 Hz; theta: 4–7,8 Hz; alpha: 7,9 -12.87 Hz; beta: 12,88 - 32 Hz. Total Power (TP) was the sum of Absolute Power (AP in μV2) of the four bands in each channel. AP of each component band from each channel was standardized by computing its Relative Power (RP), where RP = AP / TP, for Eye-open and Eye-closed Conditions. RP (×) relative to each band was log transformed into LRP = log[×/(1 – ×)] to achieve a gaussian distribution of data [27]. LRP indicates also the power of each component relative to all the others.

Overall each patient entered the study with 48 values corresponding to the following set of variables: a) 2 Conditions of recording (Eye-open and Eye-closed), b) 6 EEG channels, c) 4 component bands for each channel. A preliminary analysis was carried out in order to reduce the number of variables which was too high to be efficiently handled. Thus, individual data entered a multivariate design of analysis of variance with repeated measures [28]. Component, condition, channel, were the within subject factors and group the between-subjects one.

The condition* component*channel*group interaction was not significant, excluding the presence of systematic changes of band activities related to the different combinations of channels, conditions and groups.

On the other hand band activity averaged throughout channels showed a significant difference between conditions, apparent in all the three groups, despite the significant lower level interactions. Since our aim was that of exploring such a difference rather than describing the topographical distribution of activity on the scalp, it was decided to start from the data of single component frequency averaged throughout channels. Thus, each patient was defined by four values of AP, RP and LRP for each Condition, each value referring to a frequency component.

RP of individual alpha activity was compared with the corresponding sum of delta and theta RPs, by computing the Alpha /Slow Wave Ratio (A/SW) [29,30] for each Condition.

Analysis of q-EEG took into account first changes in A/SW Ratio, then variations of LRP in spectral profiles. A/SW differences among groups were analysed by Kruskal-Wallis analysis of variance and individual comparisons by Mann–Whitney U-test. A/SW differences between Conditions within group were assessed by Wilcoxon rank test. Individual LRP data were analysed by applying a repeated measure design of ANOVA [28]. Violations of ANOVA assumptions were corrected by changing the degrees of freedom according to the Greenhouse-Geisser procedure [28]. Multiple comparisons between means were based on the Tukey's HDS test [28,31]. All data analyses were performed with PASW software (version 18.0.0).

## Results

### F-VEP

6 patients from the Control group and 3 from the AD one were excluded from further analysis, presenting a "missing" component on both eyes in one Condition or both, mainly in Eye-open one. Thus, the following data refer to 30 patients, 11 Controls (6 men and 5 women) and 19 AD ones (11 men and 8 women).

Table 1 reports means and SEs of latencies observed in Control and AD patients. 9 out of 19 AD patients (47%) had a P2 latency difference lower than the normal cut-off (14,8 msec) and they will be referred to as patients affected by AD with Normal Latency (AD-NL). 10 patients (53%) presented a difference ≥14,8 msec. and they will be referred to as patients affected by AD with Abnormal Latency (AD-AL).

**Table 1:** F-VEP - Means and standard errors of P2 latency

	**CONTROLS**	**AD**
EYE-OPEN (EO)	136,7 ± 3,6	132,5 ± 3,1
EYE-CLOSED (EC)	140,4 ± 3.1	145,7 ± 3,9
EC – EO	3,7 ± 2,6	13,2 ± 2,2

Means ± Standard Errors of P2 latencies and of their difference between Eye-closed (EC) and Eye-Open (EO) Conditions. Controls are compared with all the AD patients.

Group means and SEs of P2 latencies and of P2 difference between Conditions are reported in Table 2 (see also Additional file 1: Table S1). P2 latencies did not differ among groups in Eye-open Condition (F=0,429; df: 2, 27; p=0,66). This means that the AD-AL lengthening of P2 difference was due to the selective increase of P2 in Eye-closed Condition.

**Table 2:** F-VEP - Means and standard errors of P2 latency

	**CONTROLS**	**AD-NL**	**AD-AL**
EYE-OPEN (EO)	136,7 ± 3,6	131,3 ± 4,5	133,5 ± 4,6
EYE-CLOSED (EC)	140,4 ± 3.1	137,2 ± 4,6	153,4 ± 5,1
EC – EO	3,7 ± 2,6	5,8 ± 2,3	19,9 ± 1.8

Means ± Standard Errors of P2 latencies and of their difference between Eye-closed (EC) and in Eye-Open (EO) Conditions. AD patients are split into AD-NL and AD-AL groups.

### q-EEG

#### A/SW ratios

**Figure 2:**
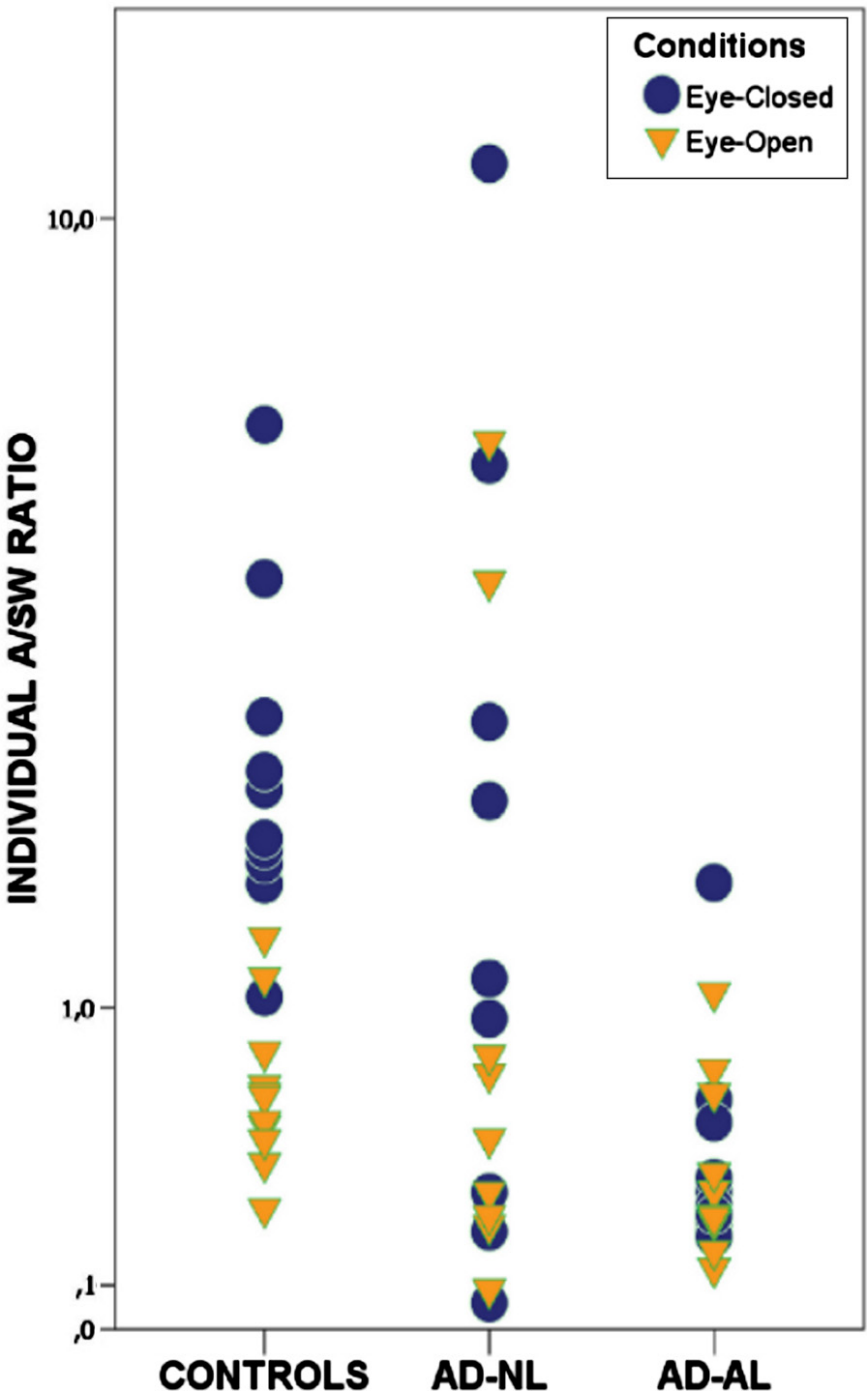
Distribution of individual A/SW Ratios as a function of Groups and Resting Conditions.

Figure 2 presents the distribution of individual Ratios within Group and Condition. Means and SEs are reported in Table 3 (see also Additional file 1: Table S2).

**Table 3:** - **Means and standard errors of A /SW ratios in different groups and rest conditions**

**GROUPS**	**EYE-OPEN**	**EYE-CLOSED**	
CONTROL	0,7 ± 0,1	2,5 ± 0,4	Z: -2,93; N=11; P<0,01
AD-NL	1,4 ± 0,7	2,7 ± 1,2	Z: -2,31; N=9 P<0,05
AD-AL	0,4 ± 0,1	0,5 ± 0,1	Z: -0.25; N= 10; ns
	K-W: 3,53; df: 2; ns	K-W: 12,12; df: 2; P<0,01	

A/SWs are averaged throughout channels. In **A**, Z-scores refer to the Wilcoxon rank test of A/SW Ratio difference between Conditions within Group. K-Ws refer to the Kruskal-Wallis Analysis of Variance of A/SW differences among Groups within Condition.

Analysis of data, in Table 3, shows that A/SW values recorded under Eye-closed Condition differed significantly from those under Eye-open one in Control group and in AD-NL one, while they did not in AD-AL patients (p=0,80).

Kruskal-Wallis analysis of variance (Table 3) shows also that, under Eye-open, A/SW values did not differ among groups, whereas they did under Eye-closed. In this Condition all the AD-AL patients, but one, had values lower than those of Controls, the difference being significant (U:2; p <0,001, one-tailed). The A/SW difference between AD-AL and AD-NL patients approached the significance (U: 25; p=0,06, one-tailed), while the AD-NL group did not differ from the Control one (U: 36; ns). All the AD-NL patients, but three, presented A/SW values within the Controls’ range.

Both the results appear clearly in Figure 2.

### Spectral profiles

Table 4 presents the group means and SEs of RPs relative to the 4 component frequencies recorded under Eye-open and Eye-closed Conditions (see also Additional file 1: Table S3).

**Table 4:** - Means and standard errors of RPs for single frequency components in different groups and rest conditions

**GROUPS**	**COMPONENTS**	**EYE-OPEN**	**EYE-CLOSED**
CONTROL	alpha	24,3 ± 2,8	44,5 ± 2,8
	beta	36,1 ± 3,9	29,4 ± 3,9
	delta	21,4 ± 2,1	10,9 ± 1,0
	theta	18,2 ± 2,2	15,2 ± 2,2
AD-NL	alpha	26,0 ± 5,2	38,6 ± 7,9
	beta	28,1 ± 2,6	23,0 ± 3,2
	delta	24,9 ± 4,5	19,8 ± 5,6
	theta	21,0 ± 2,9	18,6 ± 3,8
AD-AL	alpha	19,4 ± 3,4	20,5 ± 3,7
	beta	29,8 ± 3,8	28,4 ± 4,1
	delta	26,6 ± 3,5	28,1 ± 4,1
	theta	24,2 ± 3,4	23,0 ± 3,8

RPs are averaged throughout channels.

Individual LRP were entered into a design of analysis of variance with two within-subject variables, i.e. Component (alpha, beta, delta and theta frequency band), Condition (Eye-open and Eye-closed), and one between subject variable, i.e. Group (Control, AD-NL, AD-AL). The Condition* Component* Group interaction (Additional file 1: Table S4) was significant (F=4,45; df: 15, 13; p<0.005) allowing to split the analysis and to investigate each Conditions separately.

**Figure 3:**
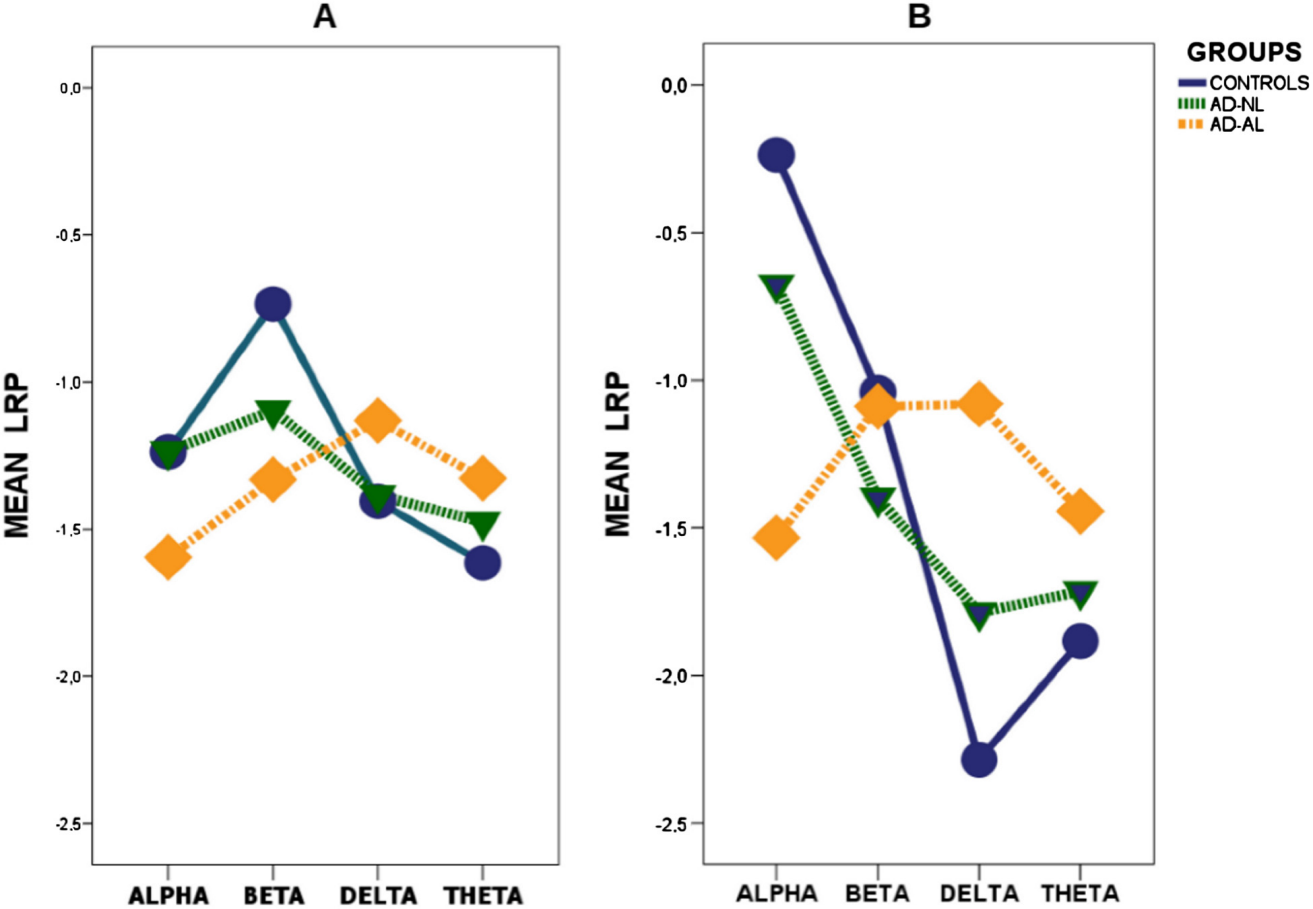
Mean spectral profile of the different Groups of patients observed in Eye-open (A) and in Eye-closed (B) Conditions.

In Eye-open Condition the Component*Group interaction was not significant, (Table 5A) indicating that spectral profiles had parallel trend (Figure 3A). LRP values did not differ among groups and components. In Eye-closed Condition the Component*Group interaction (Table 5B) was significant (p = 0,005), indicating that spectral profiles had different trends (Figure 3B) in the three groups.

**Table 5:** Analysis of Condition * Component * Group interaction

	**SS**	**df**	**MS**	**F**	**Sig. (P)**
**A.**					
**Groups** ( A )	0,05	2	0,03	0,77	0,47
Error A	0,89	27	0,03		
**Component** ( B )	2,78	3	0,93	1,87	0,14
**AB**	**3,46**	**6**	**0,58**	**1,16**	**0,34**
Error B	40,18	81	0,5		
**B.**					
**Groups** ( A )	0,06	2	0,03	1,36	0,27
Error A	0,59	27	0,02		
**Component** ( B )	16,68	3	5,56	7,06	< 0,01
**AB**	**18,14**	**6**	**3,02**	**3,84**	**< 0,01**
Error B	63,78	81	0,79		

Analysis of variance of LRPs with one between (Groups) and one within (Component) variables in Eye-Open (**A**) and in in Eye-closed (**B**) Conditions.

Individual comparison showed that the Component*Group interaction, not significant when comparing Controls and AD-NL (F=1,18; df: 3, 54; p=0,32), was highly significant in the contrast between Controls and AD-AL (F=10,73; df: 3, 57; p=0,001). In other terms the AD-NL spectral profile did not differ from that of Controls whereas the AD-AL one did (Figure 3B). In the contrast between AD-AL and AD-NL groups the Component*Group interaction approached the significance level (F=2,1; df: 3, 51; p=0,11).

Alpha band power (Figure 3B) was significantly greater in the Control Group than in AD-AL one (LRP Difference: 1,30; CI 95%: 0,38/2,21; p=0.005) while the opposite was true for delta component (LRP Difference: 1,20; CI 95%: -2,09/-0,31; p=0.006). Beta and theta components did not differ in the two groups.

## Clinical parameters of AD patients

Table 6 (see also Additional file 1: Table S5) presents the clinical data of Control, AD-NL and AD-AL patients. AD patients in the two groups did not differ from each other with respect to age, (t = 0,26; df: 17; ns), educational level (t = 0,98; df: 17; ns) and duration of the illness (t = 1,38; df: 17; ns).

**Table 6:** Clinical data

	**CONTROLS**	**AD-NL**	**AD-AL**
	**(n: 11)**	**(n: 9)**	**(n: 10)**
Age (years	67,9 ± 4,9	70,1 ± 2,7	70,7 ± 6,3
Schooling (years)	6,18 ± 1,8	5,6 ± 3,3	7,2 ± 3,9
Sex (N. women)	5	4	4
Symptom duration (months)	#	13,2 ± 2,4	14,7 ± 2,2
MMSE (corrected)	26,3 ± 1,7	18 ± 2,6	15,8 ± 3,6
DRS Verbal score (raw)	72,4 ± 9,0	43 ± 7,0	37,7 ± 7,9
DRS Non-verbal score (raw)	55,9 ± 1,8	44,6 ± 6,7	45,7 ± 7,0
DRS Total score (corrected)	0,91 ± 0,06	0,63 ± 0,05	0,592 ± 0,06

Means ± SD of clinical parameters in the 3 Groups. Patients with “missing” P2 were excluded.

AD-NL patients did not differ from AD-AL both with respect to MMSE (t = 1.51; df:17; ns) and DRS (t = 1,61; df 17; ns). The correlation coefficients between A/SW Ratios and MMSE or DRS scores were significant neither in the AD-NL group (Eye-closed: MMSE: r = 0,37; DRS: r = −0,15 – Eye-open: MMSE: r = 0,20; DRS: r = −0,21) nor in the AD-AL one (Eye-closed: MMSE: r = 0,09; DRS: r = 0,33- Eye-open: MMSE: r = 0,24; DRS: r = 0,40).

## Discussion

Our analysis aimed to determine whether F-VEP modifications might be associated to q-EEG changes in AD patients and, in this case, whether the pattern of change were the same in the whole group.

53% of our patients, the AD-AL ones, presented a change in F-VEP occurring selectively under Eye-closed Condition, as previously shown [8]. The remaining ones, i.e. AD-NL patients, had values within normal limits. In the first case the latency difference between Conditions was significant, in the second one it was not. Question was raised whether the delayed P2 latency might have been a mere artifact. Indeed, Coburn et al [32] maintained that, with Eye-open, F-VEP takes the form and the latencies of P-VEP. According to this hypothesis, our study would have compared the latency of P100 in Eye-open Condition with the longer one of P2 in Eye-closed one. If this were the case closure of eyes ought to have been followed by the lengthening of P2 latency in all our groups. Contrary wise, delayed P2 latency occurred only in the AD-AL group. All the others did not differ from normal.

By having ruled out the possibility of an artifact, the increased P2 latency can be considered an indirect sign of functional disorganization of brain activity in Resting State [33]. This view seems to be confirmed by two concomitant changes of q-EEG spectrum, a parameter apparently independent from F-VEP.

Firstly, the AD-AL group was characterized by A/SW values which, under Eye-closed, were significantly lower than those of Controls due to the collapse of alpha activity and the increase of delta component. The analysis of AD-AL spectral profile confirmed the flattening of the curve where normal peaks were hardly identifiable.

Secondly, AD-AL patients did not present the normal difference between Eye-open and Eye-closed Conditions. In our Controls, indeed, alpha activity, dropped down by about 40% from Eye-closed to Eye-open state, in keeping with other observations [34], whereas in AD-AL patients the drop was less than 6%. The opposite occurred for delta activity.

The flattening of the Eye-closed spectrum and the lack of difference between Eye-open and Eye-closed spectral profiles were present in 9 out of 10 AD-AL patients. This indicates that these results are consistent and robust enough to stand out immediately, despite the relative smallness of the group.

In all the AD-NL patients, but three, A/SW ratios were within the normal limits. The mean spectral profile recorded under Eye-closed Condition presented the same fluctuations than that of Controls and differed significantly from the one recorded under Eye-open Condition.

The association of reduced alpha and increased delta activities has been confirmed in AD by a wealth of observations [13-15]. Lesser attention has been paid to the changes in the spectral pattern of AD when comparing Eye-open Condition with Eye-closed one.

Signorino et al [16,17] studied the changes in 6,5-12 Hz band power occurring in AD by analysing the ratio between values observed in Eye-closed and Eye-open Conditions. Such a Reactivity Index was significantly lower than that of Controls, since the alpha activity recorded in AD under Eye-closed state tended to equate that of Eye-open one or to decrease below it.

Other data, derived from non-linear analysis of EEG [35], suggest that the levelling off of spectral power in Eye-closed state is not limited to the range of components explored by Signorino et al [16,17] but it extends to the whole spectrum of frequencies. Pritchard et al [36] observed that in elder Controls the Global Complexity Index obtained under Eye-closed was significantly greater than under Eye-open. The difference faded out in AD patients where the indexes were equal. Overall, the results suggested that the AD process reduces the complexity of cortical dynamics underlining EEG [13,36] by affecting the normal capacity to modulate brain activity in response to modified sensory information, such as after closure of eyes.

By showing the lack of difference between Eye-open and Eye-closed spectral profiles, our results confirm Pritchard et al.'s observation [36] but specify that changes in Resting State involve only a part, however large, of AD population.

No patient in AD group presented visual signs or symptoms of posterior cortical atrophy [37], nor severity of cognitive decline differed between our AD groups. Probably a difference between AD-NL and AD-AL patients, if any, should have been expected in attentional performances. As matter of fact, it is known that impairment in structures related to arousal and alertness significantly correlate with defects of attention [38] whose burden does not necessarily parallel severity of cognitive impairment [39]. The suggestion, however, needs further support as our study did not examine such an aspect*.*

Since F-VEP and q-EEG changes are strictly limited to Eye-closed Condition, our data support the hypothesis that behavioural states as Eye-open and Eye-closed Rest depend, at least partly, on separate system.

Such a result fits in with recent acquisitions of functional neuroanatomy which point out at different activation patterns between states [2-4,33]. In Eye-closed Rest State changing cerebral Rhythms seem to be paralleled by connectivity pattern variations [40,42]. Amplitude of EEG alpha rhythm, expression of thalamo-cortical and cortico-cortical synchronization under Eye closed Condition, is associated with changes in fMRI signal in occipital areas and in thalamus [4]. Correlated activities of medial thalamus and of anterior midbrain seem to precede the start of alpha activity [40].

The functional changes are due, at least partly, to neuronal activity [3,42] whose pattern of activation in Eye-closed Condition involves subcortical structures mostly responsible for independent modulation of alpha rhythm and F-VEP.

The impairment of such independent parameters as F-VEP and q-EEG profile under the same behavioural state, indirectly seem to support the network degeneration hypothesis according to which intrinsic connectivity networks might be the selective targets of specific neurodegenerative diseases [6,7,43]. F-VEP and Power Spectrum changes, hence, would witness the involvement of such a network system in our AD-AL patients.

In AD-NL patients, cognitive defects were not associated with changes in the two modalities. Probably the distribution of neurodegenerative process differed from that of AD-AL patients, though both groups presented the same level of dementia. These data suggest that the system damaged in AD-AL group has no effect on cognitive defects at least as they appear from measures drawn from Mental State Scales as MMSE or DRS. At the same time, the occurrence of contrasting results between F-VEP and q-EEG, mainly shown by those AD-NL patients who presented abnormal q-EEG, confirms that the two modalities are functionally independent from each other.

Summing up, F-VEP and q-EEG changes due to AD are likely to be associated to each other under Eye-closed Condition in a part of AD population. Such a group, identified by using F-VEP as a marker, seems to witness a change in the functional architecture of Resting State in Eye-closed Condition. Other patterns of change can occur, as suggested by the heterogeneity of AD population [18,44]. Whether these different patterns have distinct anatomo-clinical correlates is matter of further investigation.

## Abbreviations

AD: Alzheimer's Disease; AD-AL: Alzheimer's Disease with Abnormal Latency; AD-NL: Alzheimer's Disease with Normal Latency; F.VEP: Flash-Visual Evoked Potential; P-VEP: Pattern-Visual Evoked Potential; q-EEG: Quantitative-EEG; MMSE: Mini Mental State Examination; DRS: Dementia Rating Scale.

## Competing interests

AT, LS, FB, MM declare no competing interests.

## Authors’ contributors

AT, LS, MM, FB contributed to the conception, design and execution of the research project. All the authors were involved in preparing the draft, review and critical analysis of the manuscript. AT who carried out the statistical analysis, takes responsibility for the integrity of the data and the accuracy of the data analysis and also for approval and submission of the manuscript.

## Pre-publication history

The pre-publication history for this paper can be accessed here:

http://www.biomedcentral.com/1471-2377/12/145/prepub

## Supplementary Material

Additional file 1 **Table S1. **- F-VEP P2 latency (msec) as a function of group and condition. **Table S2** -Individual A/SW ratios averaged throughout channels as a function of condition. **Table S3** - Individual Relative Power [RP = (AP/TP)*100] averaged throughout channels as a function of condition. **Table S4** - Summary of main Analysis of Variance. **Table S5** Clinical Data. Click here for file
