# Supplementary material for: Resting state in Alzheimer's disease: a concurrent analysis of Flash-Visual Evoked Potentials and quantitative EEG
Source: BMC Neurol. 2012 Nov 28;12:145. doi: 10.1186/1471-2377-12-145 (PMC3527189; doi:10.1186/1471-2377-12-145)
Supplement: Additional file 1 — Table S1. - F-VEP P2 latency (msec) as a function of group and condition. Table S2 -Individual A/SW ratios averaged throughout channels as a function of condition. Table S3 - Individual Relative Power [RP = (AP/TP)*100] averaged throughout channels as a function of condition. Table S4 - Summary of main Analysis of Variance. Table S5 Clinical Data. [file 1471-2377-12-145-S1.doc]

**TABLE S1 - F-VEP P2 LATENCY (MSEC) AS A FUNCTION OF GROUP AND CONDITION**

| GROUP | PATIENT | EYE-OPEN | | | EYE-CLOSED | | | DIFFERENCE BETWEN MEANS |
| --- | --- | --- | --- | --- | --- | --- | --- | --- |
|  |  | OD | OS | MEAN | OD | OS | MEAN |  |
| CONTROLS | M.A. | 176 | 158 | 167 | 153 | . | 153 | -14,0 |
|  | G.AI. | 131 | 125 | 128 | 131 | 122 | 126,5 | -1,5 |
|  | M.O. | 143 | 140 | 141,5 | . | 144 | 144 | 2,5 |
|  | A.N. | 129 | 130 | 129,5 | 136 | 139 | 137,5 | 8,0 |
|  | G.N. | 142 | 146 | 144 | 150 | 160 | 155 | 11,0 |
|  | L.U. | 130 | 129 | 129,5 | 134 | 143 | 138,5 | 9,0 |
|  | O.X. | 129 | 130 | 129,5 | 125 | 120 | 122,5 | -7,0 |
|  | V.G. | 134 | 132 | 133 | 146 | 140 | 143 | 10,0 |
|  | S.O. | 139 | 145 | 142 | 145 | 140 | 142,5 | 0,5 |
|  | B.A. | 127 | 124 | 125,5 | 133 | 133 | 133 | 7,5 |
|  | G.A. | 134 | 135 | 134,5 | 150 | 148 | 149 | 14,5 |
|  | Mean | 137,6 | 135,8 | 136,7 | 140,3 | 138,9 | 140,4 | 3,7 |
|  | Median | 134 | 132 | 133 | 140,5 | 140 | 142,5 | 7,5 |
|  | S.E. | 4,2 | 3,1 | 3,6 | 2,9 | 3,6 | 3,1 | 2,6 |
| AD-NL | N.O. | 107 | 110 | 108,5 | 109 | 104 | 106,5 | -2,0 |
|  | R.U. | 135 | 132 | 133,5 | 142 | 138 | 140 | 6,5 |
|  | C.I. | . | 148 | 148 | 151 | 156 | 153,5 | 5,5 |
|  | M.E. | 145 | 148 | 146,5 | 138 | . | 138 | -8,5 |
|  | A.V. | 135 | 135 | 135 | 147 | 145 | 146 | 11,0 |
|  | L.A. | 126 | 128 | 127 | 138 | 133 | 135,5 | 8,5 |
|  | Z.U. | 146 | 141 | 143,5 | 149 | 153 | 151 | 7,5 |
|  | I.V. | 121 | 116 | 118,5 | 131 | 128 | 129,5 | 11,0 |
|  | C.O. | 121 | 122 | 121,5 | 138 | 131 | 134,5 | 13,0 |
|  | Mean | 129,5 | 131,1 | 131,3 | 138,1 | 136 | 137,2 | 5,8 |
|  | Median | 130,5 | 132 | 133,5 | 138 | 135,5 | 138 | 7,5 |
|  | S.E. | 4,4 | 4,5 | 4,5 | 4,2 | 5,5 | 4,6 | 2,3 |
| AD-AL | F.A. | 154 | 141 | 147,5 | 170 | 156 | 163 | 15,5 |
|  | O.C. | 140 | 149 | 144,5 | 166 | 179 | 172,5 | 28,0 |
|  | T.O. | 135 | 145 | 140 | 151 | 159 | 155 | 15,0 |
|  | B.A. | 103 | 111 | 107 | 130 | 121 | 125,5 | 18,5 |
|  | P.R. | 129 | 130 | 129,5 | 144 | 144 | 144 | 14,5 |
|  | A.G. | 137 | 146 | 141,5 | 167 | 176 | 171,5 | 30,0 |
|  | P.A. | 146 | 157 | 151,5 | 161 | 179 | 170 | 18,5 |
|  | C.A. | 133 | 136 | 134,5 | 151 | 149 | 150 | 15,5 |
|  | P.O. | 115 |  | 115 | 138 | 128 | 133 | 18,0 |
|  | M.A. | 125 | 125 | 125 | 150 | . | 150 | 25,0 |
|  | Mean | 131,7 | 137,8 | 133,6 | 152,8 | 154,6 | 153,5 | 19,9 |
|  | Median | 134 | 141 | 137,3 | 151 | 156 | 152,5 | 18,3 |
|  | S.E. | 4,7 | 4,9 | 4,6 | 4,2 | 6,8 | 5,1 | 1,8 |

**TABLE S2 -INDIVIDUAL A/SW RATIOS AVERAGED THROUGHOUT CHANNELS**

**AS A FUNCTION OF CONDITION**

| GROUPS |  | EYE-OPEN | EYE-CLOSED |
| --- | --- | --- | --- |
| CONTROL | M.A. | 0,66 | 1,88 |
|  | G.AI. | 0,82 | 4,05 |
|  | M.O. | 1,33 | 2,2 |
|  | A.N. | 0,3 | 2,75 |
|  | G.N. | 1,13 | 1,73 |
|  | L.U. | 0,43 | 1,82 |
|  | O.X. | 0,65 | 6,05 |
|  | V.G. | 0,5 | 2,33 |
|  | S.O. | 0,57 | 1,05 |
|  | B.A. | 0,69 | 1,63 |
|  | G.A. | 0,55 | 1,61 |
|  | Mean | 0,69 | 2,46 |
|  | SE | 0,1 | 0,4 |
|  | Median | 0,65 | 1,88 |
| Interquartile Range | | 0,31 | 1,12 |
| AD-NL | N.O. | 0,28 | 0,23 |
|  | R.U. | 4,02 | 5,46 |
|  | C.I. | 0,25 | 0,34 |
|  | M.E. | 5,79 | 11,38 |
|  | A.V. | 0,74 | 2,71 |
|  | L.A. | 0,81 | 2,13 |
|  | Z.U. | 0,09 | 0,06 |
|  | I.V. | 0,34 | 0,95 |
|  | C.O. | 0,51 | 1,13 |
|  | Mean | 1,42 | 2,71 |
|  | SE | 0,7 | 1,2 |
|  | Median | 0,51 | 1,13 |
| Interquartile Range | | 2,15 | 3,8 |
| AD-AL | F.A. | 0,35 | 0,26 |
|  | O.C. | 0,66 | 0,56 |
|  | T.O. | 0,27 | 0,28 |
|  | B.A. | 0,14 | 0,23 |
|  | P.R. | 0,28 | 0,3 |
|  | A.G. | 0,26 | 0,35 |
|  | P.A. | 0,18 | 0,22 |
|  | C.A. | 0,75 | 0,64 |
|  | P.O. | 0,4 | 0,39 |
|  | M.A. | 1,07 | 1,62 |
|  | Mean | 0,44 | 0,49 |
|  | SE | 0,1 | 0,1 |
|  | Median | 0,31 | 0,33 |
| Interquartile Range | | 0,44 | 0,33 |

**TABLE S3 - INDIVIDUAL RELATIVE POWER [RP = (AP/TP)*100] AVERAGED THROUGHOUT CHANNELS AS A FUNCTION OF CONDITION**

| **GROUPS** | Patients | **EYE-OPEN (EO)** | | | |  | **EYE-CLOSED (EC)** | | | |
| --- | --- | --- | --- | --- | --- | --- | --- | --- | --- | --- |
|  |  | **Alpha** | **Beta** | **Delta** | **Theta** |  | **Alpha** | **Beta** | **Delta** | **Theta** |
| **CONTROL** | M.A. | 18,8 | 52,7 | 18,3 | 10,2 |  | 35,0 | 42,2 | 13,4 | 9,4 |
|  | G.AI. | 31,2 | 28,0 | 19,9 | 21,0 |  | 62,3 | 15,0 | 10,3 | 12,5 |
|  | M.O. | 42,1 | 20,0 | 18,5 | 19,4 |  | 50,6 | 13,7 | 10,4 | 25,4 |
|  | A.N. | 11,9 | 41,3 | 29,3 | 17,5 |  | 37,3 | 47,2 | 10,1 | 5,3 |
|  | G.N. | 36,8 | 30,6 | 11,5 | 21,1 |  | 45,5 | 28,1 | 7,0 | 19,5 |
|  | L.U. | 21,0 | 29,6 | 23,2 | 26,1 |  | 41,6 | 23,4 | 15,2 | 19,8 |
|  | O.X. | 18,4 | 52,7 | 19,5 | 9,4 |  | 48,2 | 40,6 | 5,5 | 5,7 |
|  | V.G. | 23,3 | 25,3 | 35,7 | 15,6 |  | 54,1 | 17,0 | 14,3 | 14,5 |
|  | S.O. | 22,2 | 34,5 | 26,7 | 16,6 |  | 36,1 | 28,5 | 14,6 | 20,8 |
|  | B.A. | 27,5 | 24,8 | 14,4 | 33,4 |  | 47,2 | 21,1 | 6,7 | 25,0 |
|  | G.A. | 14,0 | 58,1 | 17,9 | 10,0 |  | 31,7 | 46,9 | 12,2 | 9,2 |
|  | Mean | 24,3 | 36,2 | 21,4 | 18,2 |  | 44,5 | 29,4 | 10,9 | 15,2 |
|  | SE | 2,8 | 3,9 | 2,1 | 2,2 |  | 2,8 | 3,9 | 1,0 | 2,2 |
|  | Median | 22,2 | 30,6 | 19,5 | 17,5 |  | 45,5 | 28,1 | 10,4 | 14,5 |
| Interquartile Range | | 12,8 | 27,4 | 8,8 | 10,9 |  | 14,4 | 25,2 | 7,3 | 11,6 |
| **AD-NL** | N.O. | 13,6 | 30,9 | 22,4 | 33,2 |  | 11,3 | 34,1 | 23,2 | 31,5 |
|  | R.U. | 51,4 | 34,4 | 4,5 | 9,6 |  | 62,7 | 24,6 | 4,2 | 8,5 |
|  | C.I. | 16,2 | 18,4 | 35,2 | 30,1 |  | 19,9 | 17,7 | 24,5 | 38,0 |
|  | M.E. | 48,7 | 34,2 | 10,7 | 6,5 |  | 69,0 | 24,7 | 2,7 | 3,6 |
|  | A.V. | 29,0 | 20,1 | 28,5 | 22,5 |  | 60,7 | 13,1 | 13,6 | 12,6 |
|  | L.A. | 30,4 | 29,9 | 18,8 | 21,0 |  | 52,5 | 22,4 | 10,7 | 14,5 |
|  | Z.U. | 6,5 | 17,8 | 51,0 | 24,8 |  | 5,1 | 7,0 | 59,3 | 28,6 |
|  | I.V. | 14,8 | 39,4 | 29,6 | 16,2 |  | 28,7 | 37,3 | 22,1 | 11,9 |
|  | C.O. | 23,4 | 28,0 | 23,7 | 24,8 |  | 37,4 | 26,2 | 17,8 | 18,7 |
|  | Mean | 26,0 | 28,1 | 24,9 | 21,0 |  | 38,6 | 23,0 | 19,8 | 18,6 |
|  | SE | 5,2 | 2,6 | 4,5 | 2,9 |  | 7,9 | 3,2 | 5,6 | 3,8 |
|  | Median | 23,4 | 29,9 | 23,7 | 22,5 |  | 37,4 | 24,6 | 17,8 | 14,5 |
| Interquartile Range | | 25,3 | 15,1 | 17,7 | 14,5 |  | 46,1 | 14,7 | 16,4 | 19,9 |
| **AD-AL** | F.A. | 17,9 | 29,1 | 34,7 | 18,3 |  | 15,8 | 22,3 | 48,9 | 12,9 |
|  | O.C. | 21,7 | 35,4 | 7,1 | 35,9 |  | 20,2 | 32,5 | 7,0 | 40,3 |
|  | T.O. | 13,8 | 31,8 | 16,4 | 38,1 |  | 15,6 | 26,9 | 19,4 | 38,1 |
|  | B.A. | 7,6 | 35,6 | 44,5 | 12,4 |  | 12,0 | 34,5 | 45,0 | 8,5 |
|  | P.R. | 10,3 | 46,3 | 32,7 | 10,8 |  | 10,9 | 48,0 | 28,3 | 12,8 |
|  | A.G. | 17,9 | 13,2 | 36,4 | 32,5 |  | 22,5 | 10,7 | 37,4 | 29,4 |
|  | P.A. | 9,4 | 43,8 | 29,6 | 17,3 |  | 11,0 | 42,6 | 31,9 | 14,5 |
|  | C.A. | 28,4 | 33,2 | 21,8 | 16,6 |  | 23,9 | 38,3 | 22,1 | 15,7 |
|  | P.O. | 23,1 | 16,5 | 23,3 | 37,2 |  | 22,3 | 15,6 | 24,2 | 37,9 |
|  | M.A. | 44,1 | 13,3 | 19,4 | 23,2 |  | 50,8 | 13,0 | 16,4 | 19,9 |
|  | Mean | 19,4 | 29,8 | 26,6 | 24,2 |  | 20,5 | 28,4 | 28,1 | 23,0 |
|  | SE | 3,4 | 3,8 | 3,5 | 3,4 |  | 3,7 | 4,1 | 4,1 | 3,9 |
|  | Median | 17,9 | 32,5 | 26,4 | 20,7 |  | 18,0 | 29,7 | 26,2 | 17,8 |
| Interquartile Range | | 14,4 | 21,9 | 16,5 | 20,6 |  | 11,1 | 24,4 | 20,7 | 25,0 |

**TABLE S4 - SUMMARY OF MAIN ANALYSIS OF VARIANCE**

**TABLE S5 – CLINICAL DATA**

| GROUPS | PATIENTS | SEX | AGE | SCHOOL (Y) | MMSE-c | RVS | RNVS | RTS | CTS |
| --- | --- | --- | --- | --- | --- | --- | --- | --- | --- |
| CONTROLS | M.A. | 1 | 61 | 8 | 28 | 80 | 56 | 136 | 0,93 |
|  | G.AI. | 1 | 63 | 8 | 27 | 82 | 57 | 139 | 0,96 |
|  | M.O. | 1 | 68 | 8 | 26 | 74 | 56 | 130 | 0,9 |
|  | A.N. | 2 | 71 | 8 | 27,4 | 68 | 58 | 126 | 0,88 |
|  | G.N. | 1 | 73 | 5 | 23,3 | 69 | 53 | 122 | 0,89 |
|  | L.U. | 1 | 71 | 5 | 28,3 | 76 | 57 | 133 | 0,96 |
|  | O.X. | 2 | 61 | 3 | 25,4 | 71 | 57 | 128 | 0,92 |
|  | V.G. | 2 | 73 | 5 | 23,3 | 56 | 53 | 109 | 0,79 |
|  | S.O. | 2 | 64 | 5 | 25,9 | 59 | 55 | 114 | 0,81 |
|  | B.A. | 1 | 68 | 8 | 27 | 85 | 55 | 140 | 0,97 |
|  | G.A. | 2 | 74 | 5 | 27,3 | 76 | 58 | 134 | 0,97 |
|  | mean |  | 67,9 | 6,2 | 26,3 | 72,4 | 55,9 | 128,3 | 0,91 |
|  | SD |  | 4,9 | 1,8 | 1,7 | 9 | 1,8 | 10 | 0,06 |
| AD-NL | N.O. | 1 | 67 | 12 | 17 | 48 | 53 | 101 | 0,67 |
|  | R.U. | 1 | 70 | 5 | 15,3 | 39 | 45 | 84 | 0,62 |
|  | C.I. | 2 | 68 | 5 | 15,9 | 28 | 44 | 72 | 0,53 |
|  | M.E. | 2 | 67 | 5 | 21 | 47 | 37 | 84 | 0,61 |
|  | A.V. | 1 | 72 | 3 | 20,7 | 45 | 44 | 89 | 0,67 |
|  | L.A. | 2 | 71 | 3 | 21,7 | 40 | 52 | 92 | 0,69 |
|  | Z.U. | 1 | 70 | 5 | 19,3 | 47 | 46 | 93 | 0,68 |
|  | I.V. | 2 | 73 | 10 | 15,4 | 41 | 48 | 89 | 0,61 |
|  | C.O. | 1 | 73 | 2 | 15,7 | 52 | 32 | 84 | 0,65 |
|  | mean |  | 70,1 | 5,6 | 18 | 43 | 44,6 | 87,6 | 0,64 |
|  | SD |  | 2,4 | 3,3 | 2,7 | 7 | 6,7 | 8 | 0,05 |
| AD-AL | F.A. | 1 | 69 | 8 | 21 | 41 | 45 | 86 | 0,6 |
|  | O.C. | 1 | 77 | 10 | 19,7 | 39 | 55 | 94 | 0,65 |
|  | T.O. | 2 | 69 | 8 | 15 | 36 | 48 | 84 | 0,59 |
|  | B.A. | 1 | 71 | 4 | 14,7 | 30 | 47 | 77 | 0,58 |
|  | P.R. | 1 | 68 | 17 | 19,8 | 54 | 56 | 110 | 0,69 |
|  | A.G. | 2 | 79 | 5 | 10,7 | 28 | 32 | 60 | 0,47 |
|  | P.A. | 1 | 73 | 5 | 10,3 | 31 | 40 | 71 | 0,53 |
|  | C.A. | 1 | 71 | 5 | 14,3 | 33 | 45 | 78 | 0,58 |
|  | P.O. | 2 | 56 | 5 | 15,9 | 40 | 47 | 87 | 0,61 |
|  | M.A. | 2 | 74 | 5 | 16,3 | 45 | 42 | 87 | 0,64 |
|  | mean |  | 70,7 | 7,2 | 15,8 | 37,7 | 45,7 | 83,4 | 0,59 |
|  | SD |  | 6,3 | 3,9 | 3,6 | 7,9 | 7 | 13,4 | 0,06 |
|  |  |  |  |  |  |  |  |  |  |
| MMSEc | MMSE score corrected for age and educational level | | | | | | | | |
| MDRS | **VS**: Verbal Score; **NVS**: Non Verbal score; **TS**: MDRS Total Score (VS + NVS); **CTS**: Total Score corrected for age and educational level | | | | | | | | |
